# Energy restriction and iron supplementation improve iron status in women with obesity regardless of red meat consumption: a randomized controlled trial

**DOI:** 10.1038/s41598-026-53056-8

**Published:** 2026-05-21

**Authors:** Salvador Ortiz-Gutiérrez, Aurora E. Serralde-Zúñiga, Luis E. González-Salazar, M. Guadalupe Estrada-Trujillo, Adriana Flores-Lopéz, Karla G. Hernández-Gómez, Edgar Pichardo-Ontiveros, Andrea Díaz-Villaseñor, Azalia Ávila-Nava, Natalia Vázquez-Manjarrez, Mariana Villegas-Romero, Rocio Guizar-Heredia, Angélica Borja-Magno, Berenice Palacios-González, Héctor Infante-Sierra, Elena Tuna-Aguilar, Laura A. Velázquez-Villegas, Ana Vigil-Martínez, Ana Ligia Gutiérrez-Solis, Brenda Pacheco-Hernández, Isabel Medina-Vera, Adriana M. López-Barradas, Emma Chávez-Manzanera, J. Gerardo Reyes-García, Nimbe Torres, Armando R. Tovar, Martha Guevara-Cruz

**Affiliations:** 1 Departamento de Fisiología de la Nutrición, Instituto Nacional de Ciencias Médicas y Nutrición Salvador Zubirán (INCMNSZ), Vasco de Quiroga No. 15 Belisario Domínguez Secc. 16, Tlalpan, Ciudad de México, 14080 México; 2 Sección de Estudios de Posgrado e Investigación, Escuela Superior de Medicina, Instituto Politécnico Nacional (IPN), Ciudad de México, México; 3 Servicio de Nutriología Clínica, Instituto Nacional de Ciencias Médicas y Nutrición Salvador Zubirán, Ciudad de México, México; 4 Instituto de Investigaciones Biomédicas, Departamento de Medicina Genómica y Toxicología Ambiental, Universidad Nacional Autónoma de México (UNAM), Ciudad de México, México; 5 Unidad de Investigación, Hospital Regional de Alta Especialidad de la Península de Yucatán, Servicios de Salud del Instituto Mexicano del Seguro Social para el Bienestar (IMSS-Bienestar), Mérida, Yucatán, México; 6 Laboratorio de Envejecimiento Saludable, Centro de Investigación sobre Envejecimiento (CIE-CINVESTAV), Instituto Nacional de Medicina Genómica, Ciudad de México, México; 7 Subdirección Médica, Instituto de Seguridad y Servicios Sociales de los Trabajadores del Estado (ISSSTE), Ciudad de México, México; 8 Departamento de Hematología y Oncología, Instituto Nacional de Ciencias Médicas y Nutrición Salvador Zubirán, Ciudad de México, México; 9 Departamento de Metodología de la Investigación, Instituto Nacional de Pediatría, Ciudad de México, México; 10 Clínica de Obesidad y Trastornos de la Conducta Alimentaria, Departamento de Endocrinología y Metabolismo, Instituto Nacional de Ciencias Médicas y Nutrición Salvador Zubirán, Ciudad de México, México

**Keywords:** Iron deficiency, Obesity, Red meat, Heme iron, Dietary intervention, Biochemistry, Biomarkers, Diseases, Health care, Medical research

## Abstract

Iron deficiency without anemia (IDNA) may be linked to obesity-related inflammation. Weight reduction and heme-iron intake from red meat are proposed to improve iron status. This randomized controlled trial (NCT06104800) assessed the impact of a low-calorie, high-protein diet with red meat (RM) compared to a low-calorie, high-protein diet without red meat (WR) over 60 days on iron concentrations in women with obesity and IDNA receiving oral iron supplementation. Fifty-two females were randomized (26 per group); 45 completed the study. After intervention, serum iron increased for both groups: RM 55.8 ± 27.0 µg/dL to 69.8 ± 30.5 µg/dL, *p* = 0.05; WR 65.3 ± 28.4 µg/dL to 76.8 ± 45.6 µg/dL, *p* = 0.27. However, group-by-time analysis showed no significant differences between dietary interventions (*p* = 0.55). Additional significant improvements (*p* < 0.05) in secondary and exploratory outcomes included ferritin, transferrin, unsaturated iron-binding capacity, uric acid, and fat mass. In conclusion, no statistically significant benefit of red meat consumption over other dietary iron sources, when combined with oral iron supplementation, was demonstrated for improving iron status in women with obesity and IDNA.

**Supplementary Information:**

The online version contains supplementary material available at 10.1038/s41598-026-53056-8.

## Introduction

Iron plays a fundamental role in the proper functioning of the body and in the onset and development of diseases. Therefore, it is crucial to control the absorption, transport, storage, and use of iron to maintain homeostasis^1^. Iron deficiency (ID) occurs when iron intake is insufficient to meet requirements or to compensate for physiological or pathological losses^2,3^. This deficiency affects vulnerable groups, such as women of reproductive age, especially in low- and middle-income regions^4^, and evidence has shown that ID without anemia (IDNA) is strongly and independently associated with all-cause mortality^5^. Currently, it is not recommended to assess iron homeostasis solely by hemoglobin concentration, but rather by integrating multiple indicators, including ferritin, mean corpuscular volume (MCV), serum iron, hepcidin, or transferrin saturation^6^. In this way, ID, especially IDNA, could be promptly identified, which is relevant, as this problem is often underdiagnosed^7^. Additionally, a relationship has been described between obesity and ID, as excess adipose tissue disrupts normal iron homeostasis^8,9^. Inflammatory states can cause a functional ID by sequestering iron into cells via overexpression of hepcidin, leading to ID despite adequate iron stores^10–14^. This process may explain the link between ID and obesity, as adipose tissue secretes proinflammatory cytokines such as interleukin-6 (IL-6), thereby promoting inflammation; however, the exact underlying mechanism remains unclear^15–17^.

In this regard, dietary interventions could be a strategy to prevent or reduce cases of IDNA. Dietary iron exists as heme and non-heme iron, with different bioavailability because of their distinct absorption mechanisms. Heme iron has an absorption rate between 15% and 35%^18^, and it is found predominantly in meat as part of hemoglobin and myoglobin; red meat is the most significant source of heme iron. Non-heme iron is present in animal, plant, and iron-fortified foods, and its absorption is approximately 10% or less^18,19^. Heme and non-heme iron delivery from cells to the circulation depends on ferroportin (FPN), while heme iron has additional release mechanisms due to the presence of the heme group^18–20^. When inflammatory processes increase hepcidin, FPN function is impaired, causing non-heme iron to accumulate within tissues; however, heme iron may be less affected by hepcidin due to differences in flow pathways^21^. Consequently, it has been suggested that higher intake of heme iron sources, such as red meat, may help improve iron status when absorption is compromised by inflammation, which can occur in obesity^9^. Moreover, weight reduction through dietary interventions may help restore iron status by reducing inflammation; however, few studies have investigated this hypothesis^22^. Therefore, this study aimed to assess the impact of a low-calorie, high-protein diet that included red meat (RM) compared to a low-calorie, high-protein diet without red meat (WR) on serum iron concentrations in women with obesity and IDNA receiving oral iron supplementation.

## Methods

### Study design

**Fig. 1:**
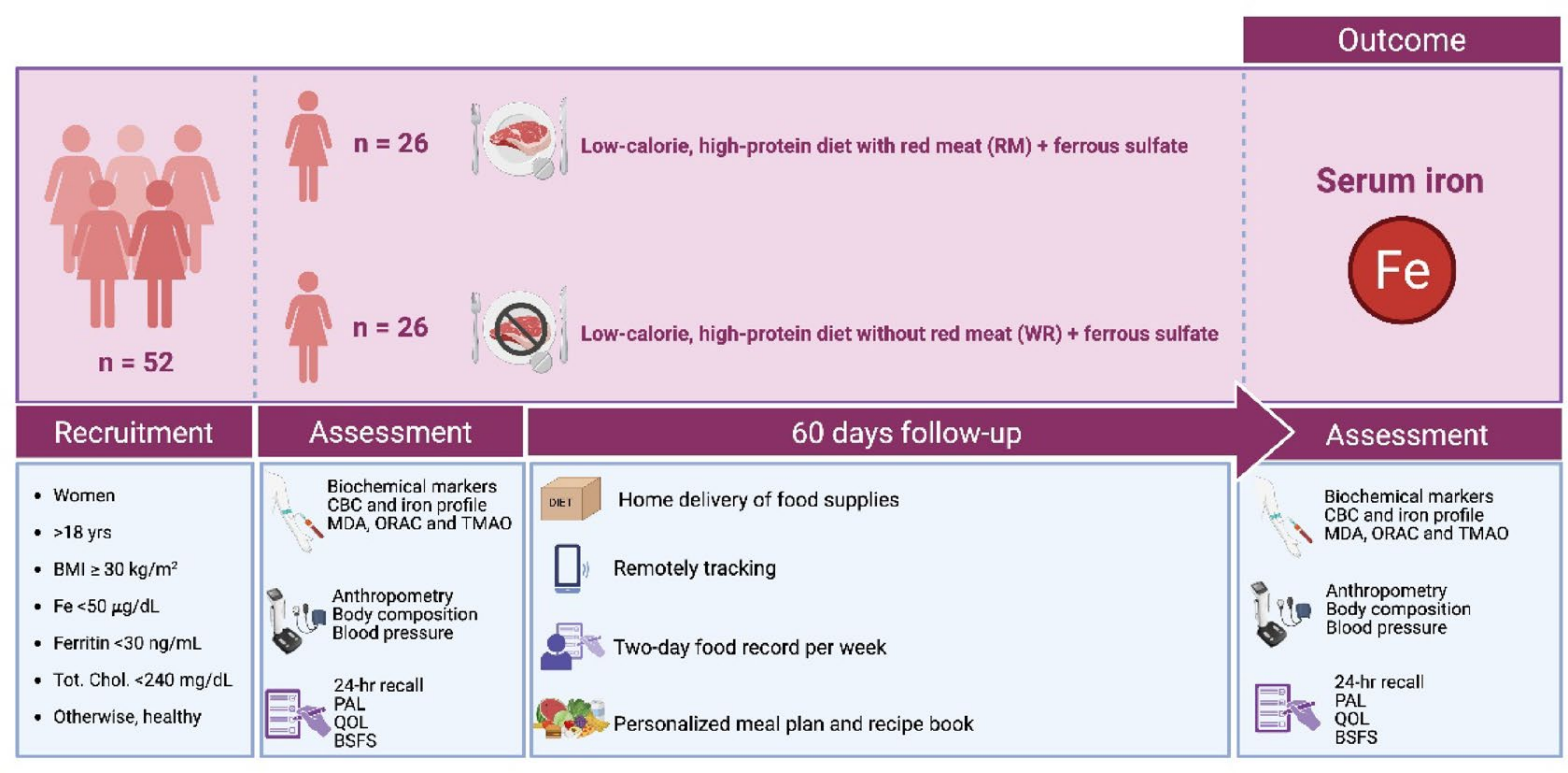
Study design. Randomized controlled trial to test the impact of two dietary interventions (RM vs. WR) plus oral iron supplementation over 60 days on iron concentrations in women with obesity and IDNA. Participants were provided with a personalized meal plan and received weekly home deliveries of all necessary food supplies tailored to their assigned diet. Abbreviations: BMI, body mass index; BSFS, Bristol Stool Form Scale; CBC, complete blood count; Fe, serum iron; IDNA, iron deficiency without anemia; MDA, malondialdehyde; ORAC, oxygen radical absorbance capacity; PAL, physical activity level; QOL, quality of life; TMAO, trimethylamine N-oxide; Tot. Chol., total cholesterol; 24-hr recall, 24-hour dietary recall. Created at https://BioRender.com.

A single-center, randomized controlled trial was conducted at the Instituto Nacional de Ciencias Médicas y Nutrición Salvador Zubirán (INCMNSZ) in Mexico City from April 2024 to January 2025 to evaluate the influence of two dietary interventions on iron status in women with obesity and IDNA. (Fig. 1) At baseline and after 60 days, iron status, biochemical markers, anthropometry, body composition, diet, and other clinical variables were assessed. The primary outcome was the change in serum iron concentrations after two months of intervention, while changes in ferritin, unsaturated iron-binding capacity (UIBC), transferrin, and hepcidin were considered as secondary outcomes; additional variables were exploratory. The primary outcome was amended from the original (change in hepcidin); the decision was based on the observations of the baseline inflammatory profile of participants. This change was reviewed and approved by the ethics committee prior to statistical analysis. The study protocol was registered on ClinicalTrials.gov (https://clinicaltrials.gov/study/NCT06104800) on October 18, 2023. This trial was conducted and reported in accordance with the CONSORT guidelines^23^.

### Study participants

Women aged 18 years or older who had obesity (BMI ≥ 30 kg/m²)^24^ and perceived themselves as healthy were invited to participate in the study through a public poster. Exclusion criteria were any disease other than obesity; chronic medication use (including oral contraceptives); history of cardiovascular events; total cholesterol > 240 mg/dL; weight loss > 3 kg in the past three months; heavy menstruation; blood loss > 500 mL from any cause; smoking (> 20 pack-years); excessive alcohol or recreational drugs consumption; pregnancy; lactation; and following a vegetarian, vegan, or plant-based diets. Participants were screened to identify IDNA, defined as a serum iron concentration < 50 µg/dL^25^ and/or serum ferritin concentration < 30 ng/mL^6^, without alteration in hemoglobin. Ferritin values were adjusted for inflammation using C-reactive protein (CRP) according to the BRINDA method^26^. The study was conducted in accordance with the principles of the Declaration of Helsinki, and all procedures involving human participants were approved by the INCMNSZ research and ethics committees (REF. 4686). All participants provided written informed consent before enrolment.

### Intervention

At baseline, participants were randomly assigned to one of two dietary groups for a two-month intervention. The RM group followed a low-calorie, high-protein diet that included unprocessed red meat (beef), while the WR group followed a low-calorie, high-protein diet with other animal protein sources, excluding red meat; protein was evenly distributed over three main meals (breakfast, lunch, and dinner). Both diets followed the same macronutrient distribution, with 50% carbohydrates and 25–30% lipids; protein was set at 1.5 g per kilogram of ideal body weight. The full composition of the diets is provided in Supplementary Table S1. Caloric restriction was individualized for each participant based on 75% of their resting energy expenditure (REE) determined by indirect calorimetry using a Quark PFT calorimeter (Cosmed). The test was performed in the morning in a quiet, thermoneutral environment (20–25 °C) with controlled humidity (45–55%) and after 10–12 h of fasting. Participants were asked to avoid vigorous exercise, alcohol consumption, and caffeine intake for at least 12 h before the test. REE was determined with the modified Weir equation^27^. Additionally, all participants received oral iron supplementation with ferrous sulfate at a dose of 200 mg/day^28^ (providing approximately 60 mg of elemental iron); participants were instructed to take the supplement one hour before breakfast to avoid interference with food. A personalized meal plan based on REE restriction and adjusted for dietary iron inhibitors was provided, along with a recipe book to encourage consumption of the specific diet, as explained by a dietitian. During follow-up, participants received weekly home deliveries of all necessary food supplies to follow the provided recipes according to their assigned diet. Before diet allocation, participants were asked about food allergies to any food included in the dietary intervention; those with recognized or suspected allergies were excluded. A dietitian monitored participants remotely throughout the study period; during this time, participants were asked to report any potential adverse effects related to the diet or the supplement intake.

### Compliance with the intervention

Diet compliance was evaluated by three independent assessment methods. For quantitative assessment, participants were instructed to keep a two-day food record per week throughout the intervention; in addition, 24-hour dietary recalls were conducted at baseline and at the final visit by a standardized dietitian. Data collected from these methods were assessed using the Food Processor software (version 11.6.522, 2018; ESHA Research, EE. UU.) by a different dietitian. Qualitative assessment consisted of visual confirmation of protein consumption according to the assigned intervention; based on analysis performed by another dietitian of digital photographs taken by participants of each food and drink they consumed each day. Adherence to oral iron supplementation was monitored weekly via follow-up calls in which participants reported their intake, and at the end of the study via tablet counting.

### Randomization assignment

Dietary allocation was performed using block randomization. An independent individual, not involved in the research team, generated the sequence using fixed blocks of four cells. The assignments were determined by a random number table, and the resulting allocation sequence was concealed in a locked cabinet to ensure blinded assignment.

### Blinding mechanisms

The dietitians involved in dietary guidance, counseling, and monitoring of participants, as well as the personnel who programmed food delivery, were not blinded to the dietary intervention. Investigators who performed the anthropometry, body composition, biochemical, and clinical determinations, as well as data analysis, were blinded to the specific intervention assigned. Meal plans and recipe books were not visually differentiated, and participants were unaware of the differences between the interventions.

### Iron indicators and biochemical markers

After a 12-hour fast, a 5 mL blood sample was collected from each participant, and the serum was obtained and stored at −80 °C until analysis. Serum, iron, ferritin, and transferrin concentrations were determined through colorimetric, chemiluminescence, and immunoturbidimetric methods, respectively. A complete blood count, (UIBC), total iron-binding capacity (TIBC), and transferrin saturation were also determined. Concentrations of hepcidin (DHP 250, R&D Systems, Inc.; reference range: 0.079–49.4 ng/mL), insulin (80-INSHU-E01.1, ALPCO), leptin (11-LEPHU-E0, ALPCO), IL-6 (EZIL6-98 K, Millipore), and adiponectin (80-ADPHU-E01, ALPCO) were determined by ELISA kits according to the manufacturer’s specifications; samples were analyzed in the same batch in duplicate and processed in multiple plates. A Cobas C111 autoanalyzer (Roche Diagnostics, Indianapolis, IN) was used to measure serum concentrations of glucose, total cholesterol, HDL cholesterol (HDL-C), LDL cholesterol (LDL-C), triglycerides, CRP, alanine aminotransferase (ALT), aspartate aminotransferase (AST), albumin, creatinine, urea, and uric acid. Oxidative stress was assessed by measuring the concentration of malondialdehyde (MDA) in plasma using a spectrophotometric method^29^, and antioxidant activity was evaluated through a fluorescence assay of the oxygen radical absorbance capacity (ORAC)^30^. Finally, trimethylamine N-oxide (TMAO) was quantified via multiple reaction monitoring on a liquid chromatography platform coupled to a 6500 + QTRAP SelexION (AB SCIEX) equipped with a Turbo V ionization source (Framingham, MA, USA); the standard curve used for quantification was 0.0075 µM to 2 µM. The data were processed with SCIEX OS software.

### Anthropometry and body composition

Body weight, height, and waist circumference (WC) were measured, as were body composition, including skeletal muscle mass, fat-free mass, fat mass (FM), visceral fat area (VFA), and total body water. A dietitian took the measurements in a standardized manner; the participants wore light clothes and no shoes during the process. Height was measured in cm to the nearest mm using a BSM 370 stadiometer (Biospace Co. Ltd., Seoul, Republic of Korea). WC was measured with a retractable measuring tape (SECA, Hamburg, Germany); the measurement was performed three times, and then the mean was obtained and recorded. Body weight and body composition were measured using a multifrequency body composition device, InBody 970 (InBody Co., Ltd., Seoul, Republic of Korea).

### Blood pressure, physical activity, and quality of life

Blood pressure was measured using a digital sphygmomanometer (Omron, HEM-781 INT); three measurements were taken at 5-minute intervals, and the average of the last two readings was recorded. The homeostatic model assessment for insulin resistance (HOMA-IR) was calculated using the formula described elsewhere^31^. The Bristol Stool Form Scale (BSFS) was used at baseline and after follow-up to assess changes in the bowel elimination pattern. The International Long-Term Physical Activity Questionnaire (IPAQ) was used at the initial and final visits to obtain results in metabolic equivalents (METs) per week. The IPAQ is available in Spanish and has been validated for the Mexican population elsewhere^32^. Participants were advised to maintain the same level of physical activity during follow-up. To assess the impact of the dietary intervention on the quality of life (QOL) of participants, the Spanish version of the 36-Item Short Form (SF-36) Health Survey, validated for the Mexican population^33^, was administered before and after the intervention. This validated version was used for non-commercial academic research purposes.

### Sample size

The required sample size was estimated based on the study by Amato et al.^34^, considering an α error of 0.05, a power of 80%, a difference in the serum iron concentration of 17 ± 1 µg/dL (3.04 ± 0.18 µmol/L), and an expected loss of 20%, at least 42 participants (21 participants per group) were needed.

### Statistical analysis

The distribution of the variables was assessed using the Kolmogorov-Smirnov or Shapiro-Wilk test. Continuous variables are presented as means ± standard deviations or medians and interquartile ranges. Dichotomous variables are expressed as frequencies and percentages. Statistical differences between groups at baseline were explored with t-tests for independent samples or the Mann–Whitney U test. After follow-up, differences (baseline vs. final) within groups were examined using paired-samples t-tests or Wilcoxon signed-rank tests. Differences between groups, over time, and the interaction between group and time were evaluated by repeated-measures ANOVA. To address potential differences in baseline values and the risk of regression to the mean, an ANCOVA was performed with baseline serum iron concentration included as a covariate. Variables with non-normal distributions (triglycerides, insulin, leptin, adiponectin, IL-6, CRP, ALT, AST, MDA, hepcidin, and TMAO) were log-transformed, and their normality was verified using the Shapiro-Wilk test before parametric analysis. The effect size was estimated using partial eta squared (η2). Bonferroni post hoc tests were performed for significant variables. A p-value < 0.05 was considered statistically significant. For the intention-to-treat (ITT) analysis, missing data were handled by multiple imputation. The primary outcome (iron concentration) was evaluated without adjustment for multiple comparisons. The prespecified secondary variables (ferritin, UIBC, transferrin, and hepcidin) were analyzed using the Bonferroni correction to account for multiple comparisons (adjusted α = 0.0125). The additional variables were considered exploratory and are presented without adjustment to generate hypotheses for future studies. Data management and statistical analysis were performed using SPSS software (IBM Corp. Released 2023. IBM SPSS Statistics for Windows, Version 29.0.2.0, Armonk, NY: IBM Corp), and figures were generated with GraphPad Prism software (GraphPad Prism version 10.3.1, Boston, MA).

## Results

**Fig. 2:**
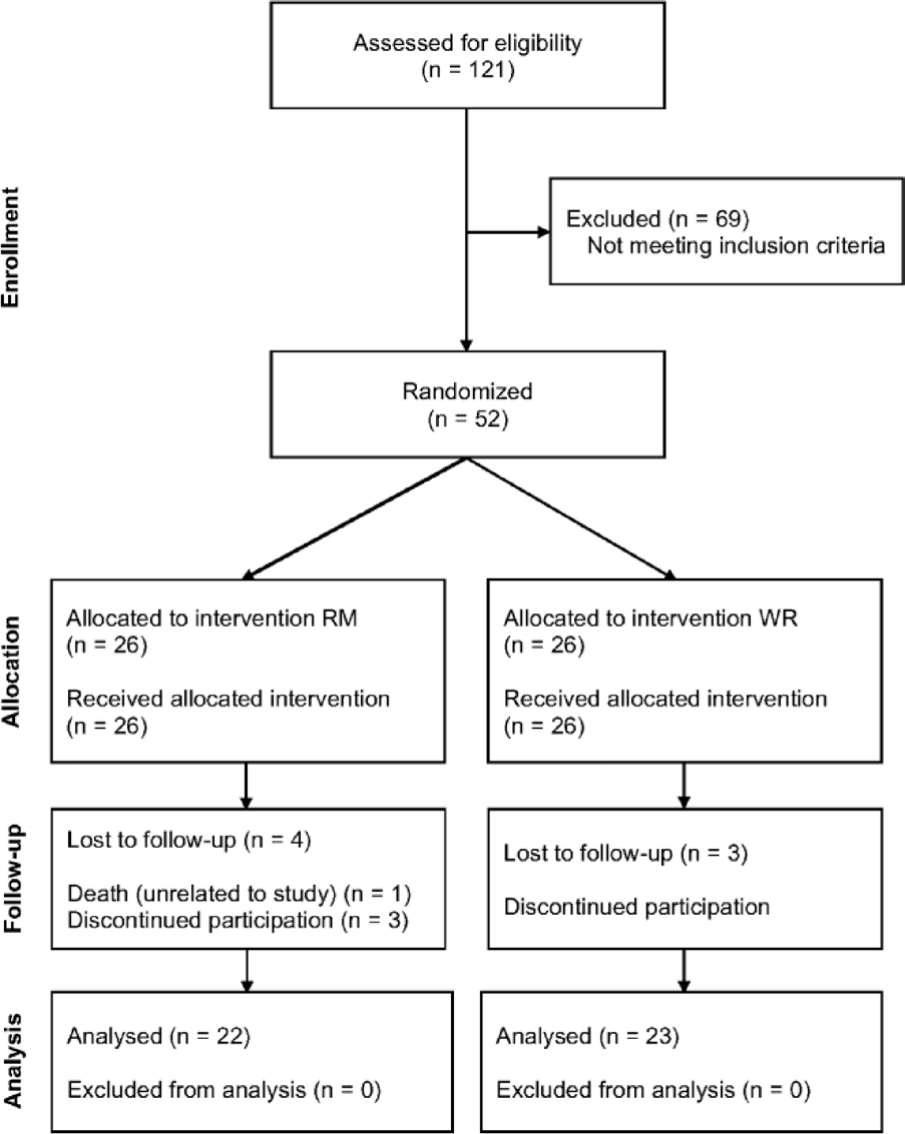
Flow chart of the study. Abbreviations: RM, low-calorie, high-protein diet with red meat plus iron supplementation; WR, low-calorie, high-protein diet without red meat plus iron supplementation.

A total of 121 women were evaluated to meet the inclusion criteria. Fifty-two volunteer women were randomized into the specific dietary intervention groups (RM or WR), 26 per group, and all received the intervention. The average age of participants was 37.3 ± 8.1 years; at baseline, there were no differences in iron indicators, biomarkers, anthropometry, body composition, or other variables between groups (Table 1). Forty-five participants completed the study (Fig. 2); no differences were observed in the percentage of diet adherence (*RM 77.3 [61.3*,* 93.1]*,* WR 84.9 [70.7*,* 96.4]; p = 0.24*) or supplementation (*RM 80.3 [77.5*,* 90.1]*,* WR 80.0 [76.9*,* 90.2]; p = 0.23*), and no adverse events associated with the interventions were reported.

**Table 1:** Baseline characteristics of participants.

	*n* = 52	RM *n* = 26	WR *n* = 26	*p*
Age (years)	37.3 ± 8.1	38.5 ± 9.1	36.1 ± 6.9	0.29
Height (cm)	158.5 ± 5.7	159.7 ± 5.7	157.4 ± 5.6	0.13
Weight (kg)	88.3 ± 12.8	88.3 ± 15.8	88.2 ± 9.2	0.97
Body mass index (kg/m^2^)	35.0 ± 4.3	34.5 ± 5.0	35.6 ± 3.6	0.36
Waist circumference (cm)	104.0 ± 10.7	103.3 ± 11.3	104.8 ± 10.3	0.61
Skeletal muscle mass (kg)	26.7 ± 3.3	26.9 ± 3.9	26.4 ± 2.7	0.59
Fat-free mass (kg)	48.3 ± 5.7	48.8 ± 6.6	47.8 ± 4.6	0.53
Fat mass (kg)	40.0 ± 9.0	39.5 ± 10.8	40.4 ± 7.0	0.72
Fat mass (%)	44.9 ± 4.8	44.3 ± 4.8	45.6 ± 4.6	0.32
Visceral fat area (cm^2^)	198.0 ± 37.0	193.4 ± 38.2	202.5 ± 35.8	0.37
Total body water (L)	35.4 ± 4.2	35.8 ± 4.9	35.0 ± 3.4	0.54
Systolic blood pressure (mm Hg)	114 ± 11	113 ± 13	114 ± 9	0.71
Diastolic blood pressure (mm Hg)	76 ± 12	74 ± 13	78 ± 10	0.17
METs (min/week)	3679 ± 2466	3632 ± 2591	3726 ± 2385	0.62
HOMA-IR	2.7 (1.9–5.3)	2.5 (1.7–5.9)	3.0 (2.2–4.3)	0.85
Glucose (mg/dL)	98.3 ± 9.8	99.5 ± 9.9	96.9 ± 9.6	0.35
Triglycerides (mg/dL)	134.3 (100.8–174.3.8.3)	122.7 (99.5–164.9.5.9)	142.6 (106.5–175.8.5.8)	0.36
Total cholesterol (mg/dL)	167.5 ± 33.4	166.4 ± 31.9	168.6 ± 35.3	0.81
LDL-C (mg/dL)	110.8 ± 28.8	110.5 ± 27.2	111.0 ± 30.7	0.94
HDL-C (mg/dL)	41.7 ± 9.2	42.2 ± 10.5	41.2 ± 7.6	0.71
Insulin (µU/mL)	11.9 8 (8.0–17.8.0.8)	10.2 (7.5–22.1)	12.5 (8.8–15.9)	0.55
Leptin (ng/mL)	57.3 (41.9–84.9)	53.4 (34.7–71.8)	60.1 (46.7–92.4)	0.13
Adiponectin (µg/mL)	17.0 (5.1–22.0)	14.0 (4.1–20.8)	18.9 (8.4–23.8)	0.27
IL-6 (pg/mL)	1.5 (0.4–2.5)	1.8 (0.2–4.0.2.0)	1.5 (0.8–2.2)	0.70
CRP (mg/L)	6.1 (3.6–11.6)	5.6 (3.1–28.6)	6.5 (3.8–10.3)	0.76
ALT (UI/L)	21.1 (17.3–27.9)	21.4 (17.5–27.8)	21.1 (16.3–30.0)	0.83
AST (UI/L)	19.3 (17.6–25.1)	19.7 (17.1–25.3)	19.1 (17.3–25.3)	0.81
Albumin (g/L)	39.5 ± 2.3	39.0 ± 2.6	39.8 ± 1.8	0.23
Creatinine (mg/dL)	0.7 ± 0.1	0.6 ± 0.1	0.7 ± 0.1	0.82
Urea (mmol/L)	4.1 ± 0.8	4.1 ± 0.8	4.2 ± 0.8	0.99
Uric acid (µmol/L)	346.5 ± 76.5	351.8 ± 75.2	341.1 ± 79.0	0.61
MDA (nmol/mL)	0.1 (0.0–1.3.0.3)	1.0 (0.0–1.6.0.6)	0.01 (0.0–1.0)	0.26
TE (µmol/mL)	3225.4 ± 854.3	3245.5 ± 929.0	3203.5 ± 786.0	0.87
TMAO (µmol/L)	1.14 (0.8–1.8)	1.44 (0.9–2.1)	1.08 (0.8–1.6)	0.35
Hemoglobin (g/dL)	14.0 ± 1.5	13.5 ± 1.8	14.4 ± 0.9	0.06
MCV (fL)	87.1 ± 8.3	86.1 ± 9.8	88.1 ± 6.5	0.38
RDW (%)	14.7 ± 1.5	15.0 ± 1.6	14.3 ± 1.4	0.11
Serum iron (µg/dL)	64.9 ± 31.0	59.9 ± 31.5	69.9 ± 30.1	0.24
UIBC (µg/dL)	270.3 ± 60.8	276.4 ± 66.3	264.2 ± 55.5	0.47
Hepcidin (ng/mL)	6.0 (1.3–7.2)	2.2 (0.9–6.7)	4.6 (2.1–8.3)	0.42
Transferrin (mg/dL)	313.7 ± 45.9	314.5 ± 48.6	312.8 ± 44.0	0.89
Ferritin (ng/mL)	24.5 ± 16.9	26.1 ± 21.2	22.9 ± 11.3	0.50
TIBC (µg/dL)	335.2 ± 47.6	336.3 ± 47.1	334.1 ± 49.0	0.87
Transferrin Saturation (%)	19.9 ± 10.1	18.6 ± 10.7	21.2 ± 9.5	0.35
Dietary energy intake (kcal/day)	1947.4 ± 749	1821.3 ± 747	2079.0 ± 744	0.15
Dietary iron intake (mg/day)	8.9 ± 5.8	8.2 ± 6.4	9.8 ± 5.1	0.10

Data are presented as the mean ± standard deviation or median (interquartile range). Statistical differences between groups were explored with the t–test for independent samples or the Mann–Whitney U test. A p-value < 0.05 was considered statistically significant.

Abbreviations: ALT, alanine transaminase; AST, aspartate transaminase; CRP, C-reactive protein; HDL-C, high-density lipoprotein cholesterol; HOMA-IR, homeostatic model assessment for insulin resistance; IL-6, interleukin-6; LDL-C, low-density lipoprotein cholesterol; MCV, mean corpuscular volume; MDA, malondialdehyde; METs, metabolic equivalent of task; RCT, randomized control trial; RDW, red cell distribution width; RM, low-calorie, high-protein diet with red meat; TE, Trolox equivalent; TIBC, total iron-binding capacity; TMAO, trimethylamine N-oxide; UIBC, unsaturated iron binding capacity; WR, low-calorie, high-protein diet without red meat.

### Primary outcome

**Fig. 3:**
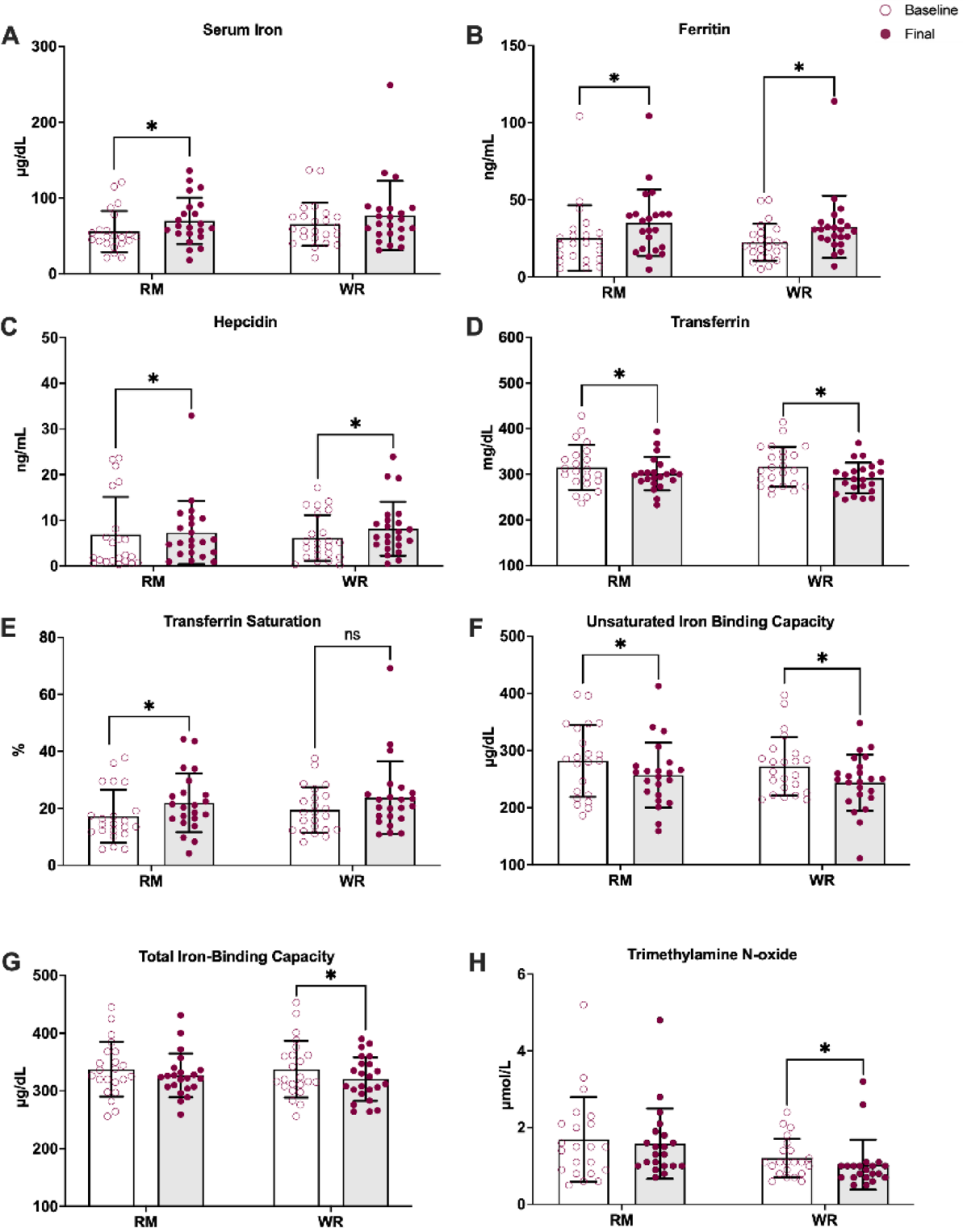
Effect of dietary intervention on iron status indicators and TMAO concentrations. Changes in concentrations of different biomarkers from baseline to the end of the study. **(A)** Serum iron (µg/dL), **(B)** Ferritin (ng/mL), **(C)** Hepcidin (ng/mL), **(D)** Transferrin (mg/dL), **(E)** Transferrin saturation (%), **(F)** Unsaturated Iron Binding Capacity (UIBC; µg/dL), **(G)** Total Iron-Binding Capacity (TIBC; µg/dL), and **(H)** Trimethylamine N-oxide (TMAO; µmol/L). Data are presented for the low-calorie, high-protein diet with red meat (RM) and the low-calorie, high-protein diet without red meat (WR) groups at baseline (empty circles) and final (filled circles). Each circle represents a participant; bars and error bars show mean ± SD. Statistical significance within groups (baseline vs. final) was assessed using a paired t-test; significant differences (*p* < 0.05) are noted with an asterisk (*).

Serum iron concentrations increased from baseline after follow-up in both groups. In the RM group, levels changed from 55.8 ± 27.0 µg/dL to 69.8 ± 30.5 µg/dL (*p < 0.05*). While in the WR group, a similar rise was noticed, from 65.3 ± 28.4 µg/dL to 76.8 ± 45.6 µg/dL, although within-group change was not statistically significant (*p = 0.27*) (Fig. 3A). Furthermore, the analysis of the interaction of group and time revealed no difference (*p* = 0.55) between the RM and WR interventions; the estimated between-group difference was small (*−2.52 µg/dL*,* 95% CI: −30.6*,* 25.6*). The ANCOVA model showed that baseline serum iron levels did not influence the values observed at the end of the study (*F [1*,* 42] = 0.098*,*p = 0.755*). Additionally, ITT analysis showed similar results (*p = 0.87*) (Supplementary Table S2).

### Secondary outcomes

#### Iron status indicators

Other changes in iron status indicators were noticed in both groups after follow-up. An increase in serum ferritin concentrations was observed (*RM: 17.9 ± 11.5 ng/mL to 26.6 ± 15.8 ng/mL*, *p* = 0.008; WR: 17.3 ± 9.9 ng/mL to 26.1 ± 18.8 ng/mL, *p* < 0.001), as well as a reduction in UIBC (*RM: 281.8 ± 62.9 µg/dL to 257.0 ± 56.8 µg/dL*, *p* < 0.001; WR: 272.3 ± 51.1 µg/dL to 243.7 ± 49.2 µg/dL, *p* < 0.001) and transferrin concentrations (*RM: 315.0 ± 49.1 mg/dL to 301.3 ± 36.4 mg/dL*, *p* = 0.04; WR: 316.7 ± 43.6 mg/dL to 291.8 ± 33.4 mg/dL, *p* < 0.001); serum hepcidin also significantly increased in both groups (*RM: 2.2 [0.9–10.4] ng/mL to 5.4 [2.8–10.4] ng/mL*, *p* = 0.03; WR: 5.0 [1.9–11.4] ng/mL to 6.5 [4.5–10.0] ng/mL, *p* = 0.02) (Fig. 3). Nevertheless, no significant group-by-time interactions were detected; therefore, the Bonferroni correction was not applied, and these results are considered exploratory and should be interpreted with caution. All participants had hemoglobin concentrations within the normal range, and no other hematological abnormalities were observed.

### Exploratory outcomes

#### Biochemical markers

After the intervention period, serum concentrations of HDL-C, uric acid, and MDA decreased in both groups. Intra-group analysis showed that the RM group had a reduction in glucose, whereas adiponectin, leptin, and TMAO decreased in the WR group. However, no interaction was observed in group-by-time analysis, indicating that such changes did not differ between interventions (Table 2).

**Table 2:** Iron status indicators and biochemical markers.

Variables	RM			WR			*p* ^b^	*p* ^c^	*p* ^d^	η^2^
	Baseline *n* = 22	Final *n* = 22	*p* ^a^	Baseline *n* = 23	Final *n* = 23	*p* ^a^				
Serum iron (µg/dL)	55.8 ± 27.0	69.8 ± 30.5	0.05	65.3 ± 28.4	76.8 ± 45.6	0.27	0.25	0.03	0.55	0.008
Ferritin (ng/mL)	17.9 ± 11.5	26.6 ± 15.8	0.008	17.3 ± 9.9	26.1 ± 18.8	< 0.001	0.99	< 0.001	0.89	0.001
UIBC (µg/dL)	281.8 ± 62.9	257.0 ± 56.8	< 0.001	272.3 ± 51.1	243.7 ± 49.2	< 0.001	0.40	< 0.001	0.40	0.01
Transferrin (mg/dL)	315.0 ± 49.1	301.3 ± 36.4	0.04	316.7 ± 43.6	291.8 ± 33.4	< 0.001	0.73	< 0.001	0.19	0.03
Hepcidin (ng/mL)	2.2 (0.9–10.4)	5.4 (2.8–10.4)	0.03	5.0 (1.9–11.4)	6.5 (4.5–10.0)	0.02	0.48	0.002	0.87	0.001
TIBC (µg/dL)	337.5 ± 47.4	326.8 ± 37.8	0.09	337.6 ± 49.1	320.5 ± 37.7	0.01	0.79	0.004	0.48	0.01
Transferrin Sat. (%)	17.2 ± 9.3	21.9 ± 10.3	0.03	19.4 ± 7.9	23.7 ± 12.7	0.10	0.26	0.008	0.62	0.006
Hemoglobin (g/dL)	13.3 ± 1.6	13.6 ± 1.0	< 0.001	14.3 ± 0.9	14.0 ± 0.8	< 0.001	0.05	< 0.001	0.06	0.08
MCV (fL)	86.0 ± 9.7	88.6 ± 5.4	< 0.001	87.5 ± 6.5	87.4 ± 4.5	< 0.001	0.39	< 0.001	0.38	0.01
RDW (%)	14.9 ± 1.5	15.2 ± 2.6	0.62	14.4 ± 1.4	14.3 ± 1.4	0.36	0.17	0.97	0.38	0.01
Glucose (mg/dL)	100.0 ± 8.2	95.5 ± 10.0	0.01	97.4 ± 10.0	96.0 ± 8.0	0.35	0.66	0.009	0.15	0.04
Triglycerides (mg/dL)	125.5 (103.1–188.6.1.6)	122.0 (106.8–164.3.8.3)	0.89	143.7 (99.9–176.0)	128.8 (97.8–169.4.8.4)	0.08	0.52	0.37	0.42	0.01
TC (mg/dL)	165.4 ± 34.5	167.4 ± 29.3	0.57	167.4 ± 37.3	166.6 ± 31.7	0.97	0.96	0.67	0.64	0.005
LDL-C (mg/dL)	110.0 ± 29.5	115.3 ± 24.0	0.11	109.8 ± 32.5	115.9 ± 29.9	0.16	0.93	0.03	0.98	0.001
HDL-C (mg/dL)	40.8 ± 9.7	40.2 ± 8.4	< 0.001	40.3 ± 7.0	36.7 ± 5.5	< 0.001	0.81	< 0.001	0.83	0.001
Insulin (µU/mL)	11.1 (7.9–26.7)	13.1 (6.9–22.4)	0.63	12.0 (8.8–15.0)	15.5 (6.7–26.0)	0.30	0.88	0.95	0.41	0.01
Leptin (ng/mL)	53.4 (34.7–71.8)	50.4 (27.2–71.6)	0.24	60.1 (46.7–92.4)	42.0 (35.9–66.3)	< 0.001	0.20	0.005	0.06	0.08
Adiponectin (µg/mL)	14.0 (4.1–20.8)	14.3 (5.2–16.7)	0.50	18.9 (8.4–23.8)	16.5 (5.3–20.5)	0.01	0.41	0.03	0.14	0.06
IL-6 (pg/mL)	1.8 (0.2–4.0.2.0)	0.8 (0.2–3.1)	0.17	1.6 (0.9–2.2)	1.2 (0.7–2.0.7.0)	0.07	0.39	0.07	0.34	0.02
CRP (mg/L)	5.3 (3.1–10.1)	5.5 (2.1–13.0)	0.70	6.5 (3.9–10.1)	7.4 (2.9–9.6)	0.39	0.69	0.42	0.88	0.001
ALT (UI/L)	21.7 (17.7–28.8)	22.8 (17.1–31.2)	0.85	22.6 (16.0–35.3.0.3)	20.4 (16.2–26.1)	0.38	0.21	0.66	0.51	0.01
AST (UI/L)	19.7 (15.8–25.3)	18.4 (16.2–23.8)	0.90	19.0 (17.6–25.3)	17.5 (15.3–21.9)	0.37	0.36	0.48	0.70	0.003
Albumin (g/L)	39.2 ± 2.7	39.3 ± 3.4	0.87	39.7 ± 1.9	38.7 ± 3.4	0.20	0.94	0.44	0.32	0.02
Creatinine (mg/dL)	0.6 ± 0.1	0.7 ± 0.1	< 0.001	0.7 ± 0.1	0.7 ± 0.2	< 0.001	0.11	< 0.001	0.23	0.03
Urea (mmol/L)	4.2 ± 0.8	4.3 ± 1.0	0.66	4.2 ± 0.9	4.6 ± 1.0	0.13	0.53	0.15	0.41	0.01
Uric acid (µmol/L)	354.0 ± 81.7	292.2 ± 82.3	0.005	348.9 ± 78.9	299.4 ± 84.3	0.005	0.96	< 0.001	0.62	0.006
MDA (nmol/mL)	1.0 (0–1.7.7)	0.01 (0–0.02.02)	0.03	0.1 (0–1.2.2)	0.01 (0–0.5.5)	0.02	0.03	0.001	0.24	0.11
TE (µmol/mL)	3243 ± 972	3632 ± 1042	0.10	3206 ± 768	3431 ± 856	0.36	0.58	0.07	0.63	0.005
TMAO (µmol/L)	1.4 (0.8–2.1)	1.4 (0.9–1.9)	0.77	1.0 (0.8–1.6)	0.8 (0.6–1.0.6.0)	0.03	0.01	0.13	0.06	0.07

Data are presented as the mean ± standard deviation or median (interquartile range). Statistical differences (baseline vs. final) within groups were examined using paired-samples t-tests or Wilcoxon signed-rank tests. Differences between groups, over time, and the interaction between group and time were evaluated by repeated-measures ANOVA. Variables with nonparametric distributions were normalized with log transformation before repeated-measures ANOVA. The effect size was estimated using partial eta squared (η^2^). A p-value < 0.05 was considered statistically significant.

Abbreviations: ALT, alanine transaminase; AST, aspartate transaminase; CRP, C-reactive protein; HDL-C, high-density lipoprotein; LDL-C, low-density lipoprotein; MCV, mean corpuscular volume; MDA, malondialdehyde; RDW, red cell distribution width; RM, low-calorie, high-protein diet with red meat; TC, total cholesterol; TE, Trolox equivalent; TIBC, total iron-binding capacity; TMAO, trimethylamine N-oxide; Transferrin Sat., transferrin saturation; UIBC, unsaturated iron binding capacity; WR, low-calorie, high-protein diet without red meat.

^a^paired-samples t-test or Wilcoxon signed-rank test.

^b^Repeated-measures ANOVA (group).

^c^Repeated-measures ANOVA (time).

^d^Repeated-measures ANOVA (group-time interaction; primary hypothesis).

### Anthropometry, body composition, diet, and other variables

After follow-up, body weight, BMI, WC, FM, and VFA decreased, with no differences between group interventions, except for FM, which was significantly lower in the WR group (*RM: 40.4 ± 11.4 to 38.9 ± 11.6 kg vs. WR: 41.4 ± 6.8* to *38.4 ± 6.9 kg; p = 0.03*) after group-by-time analysis (Table 3). After intervention, significant reductions were detected in energy intake (*RM: 1824 ± 763 kcal to 1425 ± 475 kcal*, *p* = 0.05; WR: 2079 ± 744 kcal to 1498 ± 392 kcal, *p* < 0.003) and dietary cholesterol (*RM: 251 ± 140 mg to 172 ± 82 mg*, *p* = 0.02; WR: 260 ± 126 mg to 199 ± 74 mg, *p* = 0.04), as well as increases in fiber (*RM: 0.9 ± 1.5 g to 3.2 ± 3.5 g*, *p* = 0.009; WR: 1.7 ± 2.4 g to 3.1 ± 3.2 g, *p* = 0.04) and percentage of protein consumed (*RM: 18 ± 8 to 22 ± 8*, *p* = 0.04; WR: 19 ± 5 to 22 ± 5, *p* = 0.04) per day; without differences between intervention groups (Supplementary Table S3). Systolic (SBP) and diastolic blood pressure (DBP) levels decreased from baseline to the end of the study; nevertheless, this change was significant only within the WR group (SBP: *114 ± 10 mm Hg to 107 ± 9 mm Hg*, *p* = 0.001; DBP: 78 ± 10 mm Hg to 74 ± 8 mm Hg, *p* = 0.05), without differences when interventions were compared by group-by-time analysis. HOMA-IR and BSFS scores did not significantly change within or between groups after follow-up. Additionally, a significant reduction in METs was observed after follow-up for both groups (*RM: 3680 ± 2701 min/week to 3428 ± 1907 min/week*, *p* < 0.001; WR: 3686 ± 2222 min/week to 2926 ± 1828 min/week, *p* < 0.001), regardless of the intervention. QOL improved within the RM group (*70.2 ± 17.7 to 75.3 ± 16.8*, *p* = 0.01); again, no significant group-by-time interaction was observed, indicating that the change did not differ between interventions (Table 3).

**Table 3:** Anthropometry, body composition, and other variables.

Variables	RM, *n* = 22			WR, *n* = 23			*p* ^b^	*p* ^c^	*p* ^d^	η^2^
	Baseline	Final	*p* ^a^	Baseline	Final	*p* ^a^				
Weight (kg)	88.4 ± 17	86.8 ± 16.8	< 0.001	88.8 ± 9.5	85.9 ± 9.3	< 0.001	0.94	< 0.001	0.14	0.05
BMI (kg/m^2^)	34.9 ± 5.3	34.2 ± 5.3	< 0.001	36.0 ± 3.6	34.9 ± 3.9	< 0.001	0.49	< 0.001	0.20	0.03
WC (cm)	103 ± 12	101 ± 12	0.002	106 ± 11	103 ± 10	0.01	0.51	< 0.001	0.38	0.01
SMM (kg)	26.5 ± 3.8	26.4 ± 3.8	0.75	26.2 ± 2.8	25.9 ± 2.4	0.17	0.68	0.21	0.40	0.01
FFM (kg)	48.1 ± 6.5	48 ± 6.4	0.71	47.4 ± 4.8	47.0 ± 4.1	0.23	0.63	0.24	0.48	0.01
Fat mass (kg)	40.4 ± 11.4	38.9 ± 11.6	< 0.001	41.4 ± 6.8	38.4 ± 6.9	< 0.001	0.91	< 0.001	0.03^e^	0.10
Fat mass (%)	45.1 ± 4.3	44.1 ± 4.9	0.001	46.4 ± 4.3	44.8 ± 4.1	< 0.001	0.45	< 0.001	0.14	0.05
VFA (cm^2^)	197 ± 38	188 ± 41	< 0.001	208 ± 34	196 ± 35	< 0.001	0.39	< 0.001	0.42	0.01
TBW (L)	35 ± 5	35 ± 5	0.65	35 ± 4	34 ± 3	0.23	0.64	0.22	0.50	0.01
SBP (mm Hg)	114 ± 13	110 ± 14	0.06	114 ± 10	107 ± 9	0.001	0.65	< 0.001	0.35	0.02
DBP (mm Hg)	76 ± 12	75 ± 10	0.46	78 ± 10	74 ± 8	0.05	0.80	0.05	0.37	0.01
HOMA-IR	2.7 (1.9–6.7)	3.1 (1.8–5.5)	0.47	2.6 (2.0–4.1.0.1)	3.8 (1.5–5.9)	0.56	0.85	0.77	0.34	0.02
METs (min/week)	3680 ± 2701	3428 ± 1907	< 0.001	3686 ± 2222	2926 ± 1828	< 0.001	0.37	< 0.001	0.37	0.01
QOL	70.2 ± 17.7	75.3 ± 16.8	0.01	75.3 ± 16.8	78.6 ± 12.2	0.07	0.57	0.005	0.21	0.03
Energy (kcal/day)	1824 ± 763	1425 ± 475	0.05	2079 ± 744	1498 ± 392	0.003	0.59	0.48	0.38	0.01
Protein (g/kg_ibw_)	1.5 ± 0.6	1.8 ± 0.6	0.76	1.4 ± 0.6	1.5 ± 0.4	0.07	0.80	0.31	0.32	0.02

Data are presented as the mean ± standard deviation or median (interquartile range). Statistical differences (baseline vs. final) within groups were examined using paired-samples t-tests or Wilcoxon signed-rank tests. Differences between groups, over time, and the interaction between group and time were evaluated by repeated-measures ANOVA. Variables with nonparametric distributions were normalized with log transformation before repeated-measures ANOVA. The effect size was estimated using partial eta squared (η^2^). A Bonferroni post hoc test was performed on variables that showed significant interactions with the interventions. A p-value < 0.05 was considered statistically significant.

Abbreviations: BMI, body mass index; DBP, diastolic blood pressure; FFM, fat-free mass; HOMA-IR, homeostatic model assessment for insulin resistance; ibw, ideal body weight; METs, metabolic equivalent of task; QOL, quality of life; RM, low-calorie, high-protein diet with red meat; SBP, systolic blood pressure; SMM, skeletal muscle mass; TBW, total body water; VFA, visceral fat area; WC, waist circumference; WR, low-calorie, high-protein diet without red meat. Notes: Energy and protein refer to dietary intake.

^a^paired-samples t-test or Wilcoxon signed-rank test.

^b^Repeated-measures ANOVA (group).

^c^Repeated-measures ANOVA (time).

^d^Repeated-measures ANOVA (group-time interaction; primary hypothesis).

^e^ Bonferroni post hoc test, *p* < 0.001.

## Discussion

The present study revealed no statistically significant benefit of consuming red meat over energy restriction and ensuring adequate iron intake from other dietary sources, combined with oral iron supplementation, in improving iron status in women with obesity and IDNA.

**Fig. 4:**
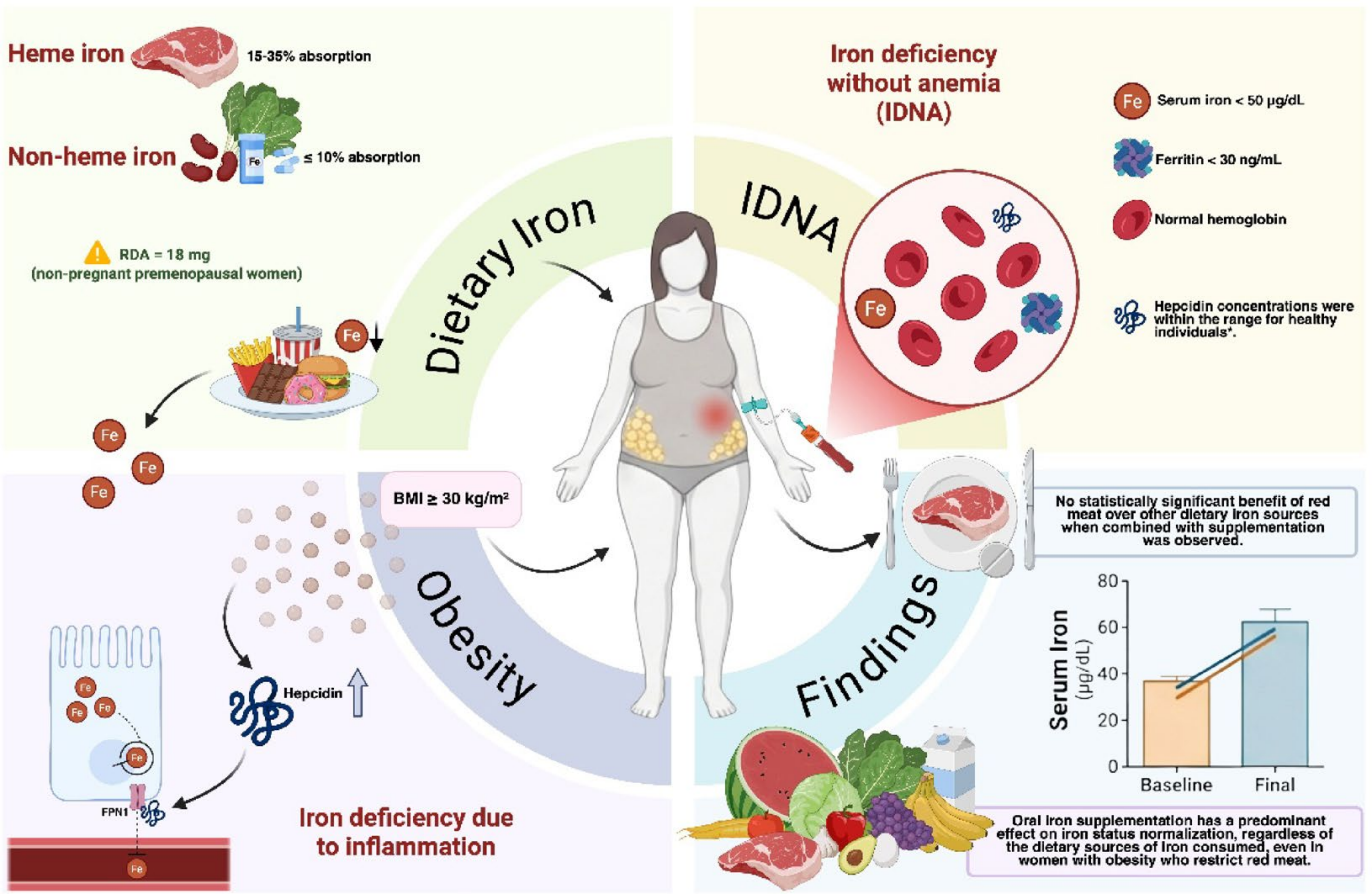
Summary of framework and findings. The left side summarizes the conceptual framework for the study, while the right side presents synthesized findings on the primary outcome and the conclusion. Dietary iron exists as heme and non-heme iron. Heme iron has an absorption rate of 15–30%, with red meat being the primary source. Non-heme iron is found in animal, plant, and iron-fortified foods, but its absorption rate is around 10% or less. The study group tends to have iron-deficient diets (8.9 mg/day), falling below the RDA (upper left). Iron delivery from cells to the circulation depends on FPN1. When inflammatory processes increase hepcidin, FPN1 function is impaired, leading to iron accumulation in tissues. This hypothesis could explain the link between IDNA and obesity (down left). In this study, IDNA was defined as a serum iron concentration < 50 µg/dL and/or a ferritin concentration < 30 ng/mL, without alterations in hemoglobin. Hepcidin concentrations were consistent with the range for healthy individuals* (upper right). After testing two dietary interventions, one with red meat and the other without, plus oral iron supplementation, no significant extra benefit was observed from red meat consumption to improve iron concentrations in women with obesity and IDNA (down right). *According to the range provided by the manufacturer of the DHP-250 assay (DHP 250, R&D Systems, Inc.; reference range: 0.079–49.4 ng/mL). Abbreviations: BMI, body mass index; FPN1, ferroportin; IDNA, iron deficiency without anemia; RDA, recommended dietary allowance. Created at https://BioRender.com.

After the intervention, no differences in iron concentration changes were observed in response to the assigned diets (RM vs. WR). Iron status improved in both groups as evidenced by increases in serum iron and ferritin concentrations and decreases in UIBC and transferrin levels. Given that both groups received identical oral iron supplementation, the findings suggest that improvements in iron status may result from supplementation and energy restriction, with no clinical advantage provided by red meat consumption, as demonstrated by the between-group analysis, concluding that both dietary patterns were effective when combined with iron supplementation (Fig. 4).

In this study, the effect of red meat consumption may be masked by iron supplementation, since both groups received ferrous sulfate (200 mg/day). On this matter, it is well documented that iron supplements change the absorption of this micronutrient in iron-depleted women who received oral supplementation; after the intake of oral iron supplements, iron absorption tends to be reduced by 35–45%, mainly due to an acute increase in hepcidin concentrations that occurs at 8 h and remains elevated for 24 h after supplement intake, as evaluated by stable iron isotope studies^35–38^. This is consistent with the higher hepcidin concentrations observed in our study for both groups after follow-up, which may be partly due to daily supplementation during the intervention period, thereby reducing the potential benefit of dietary iron. To avoid this scenario, it might have been better to use iron supplements on intermittent days, as this scheme has been shown to be effective in correcting iron deficiency, improving the bioavailability of subsequent doses, and reducing unpleasant side effects^35^.

In our study population, IDNA may be driven by distinct counterregulatory mechanisms, with coexisting insufficient dietary iron intake and obesity-related inflammation. At baseline, hepcidin concentrations were consistent with the range for healthy individuals provided by the manufacturer of the DHP-250 assay, as well as with observations in similar populations using the same analytical platform^39,40^. As mentioned, after follow-up, hepcidin concentrations increased but remained within the reference range, similar to what has been reported in some studies of women living with obesity^39–42^. This hepcidin response is likely the result of three concurrent mechanisms: first, the acute response related to daily oral iron supplementation, as described above; second, the diminished counter-regulatory suppression of hepcidin as iron is replenished, as evidenced by increases in iron and ferritin levels; and third, the upward pressure from persisting obesity-related inflammation. Iron deficiency can act as a ‘brake’ that overrides inflammatory stimuli; once this deficiency is corrected, the underlying inflammatory state may fully manifest its effect on hepcidin synthesis^43^. These complex interactions between physiological responses of iron metabolism make the link between obesity, inflammation, and hepcidin not straightforward, reflecting the heterogeneity of ID mechanisms, which are driven by the interplay of inflammatory, metabolic, hormonal, and dietary factors^40,43^.

Regarding dietary iron, the average intake at baseline was 8.9 mg/day; this amount may be insufficient compared with the recommended dietary allowances for iron in women of similar age to those in our study^44^. After intervention, there was no difference in dietary iron intake, although this result may have been overshadowed by the effect of oral supplementation, as discussed earlier. In this way, it could be important that future research consider studying a group without supplementation to have more insight into the effectiveness of dietary interventions for correcting IDNA. Furthermore, it has been documented that the sole use of iron supplements may be ineffective in the long term if dietary changes are not made to improve the dietary profile with highly bioavailable iron-rich foods^38,45^.

Overall, participants showed improvements in their diets, increasing intake of key nutrients like protein, fiber, and vitamin C, while reducing caloric intake and caffeine consumption, which may also contribute to better iron absorption. Additionally, reductions observed in fat mass, blood pressure, glucose, and uric acid may also be related to dietary changes; however, these changes may not directly affect iron status. Previous studies have reported that changes in adiposity or inflammatory markers do not necessarily translate into consistent changes in iron metabolism parameters. A study conducted in women with obesity found that iron status indicators were not different between lean and obese individuals^42^. Another important change was observed in oxidative stress, with reductions in MDA concentrations and increases in antioxidant activity after the intervention in both groups, but without group-by-time interaction; these changes might suggest an improved cardiometabolic and redox status due to better overall diet, as previously mentioned. Adopting diets higher in fruits, vegetables, legumes, and whole grains has been consistently associated with improved metabolic pathways and reduced oxidative stress^46^; vitamins, polyphenols, and other bioactive compounds contribute to antioxidant defense mechanisms and may reduce lipid peroxidation, as reflected by lower MDA^47^. In contrast, reduced intake of saturated fats has been linked to improvements in insulin sensitivity, blood pressure regulation, and body composition. Importantly, these effects appear to be driven more by the overall diet than by the inclusion or exclusion of specific food groups^48^. For the WR group, a reduction in TMAO concentrations was observed in the intra-group analysis, but no interaction with diet was found after group-by-time analysis. Although this indicates that there is no significant result to conclude that avoiding red meat reduced TMAO concentrations in our study, it aligns with existing evidence. For instance, Wang et al. reported that dietary exclusion of red meat decreased plasma TMAO after 4 weeks^49^. Similarly, the SWAP-MEAT study demonstrates a reduction in TMAO after 8 weeks of replacing red meat with a plant-based alternative^50^. Therefore, despite the lack of significance, these results could still suggest a potential clinical impact and provide additional long-term benefits, which should be highlighted, given the implications of TMAO for cardiovascular disease^51^, a common health issue among people with obesity. As for physical activity, reductions from baseline conditions were observed in both groups. It is possible that, at enrollment, participants overreported their physical activity due to social desirability bias, a common bias in lifestyle-related studies^52^. Despite this finding, no differences were observed between the intervention groups; therefore, we consider that this change did not affect the results.

Also, it is important to note that iron deficiency is closely linked to reproductive status in women due to various factors, including continuous blood loss, increased iron demands, and hormonal sensitivity, all of which can increase the risk of deficiency, particularly among premenopausal women^53^. In our study, the mean age was 37 years, so most participants were considered premenopausal; no differences in age or reproductive status were observed between groups. Therefore, conducting a stratified analysis by age category or by pre- or postmenopausal status was not feasible. Future research on interventions to address iron deficiency should take these differences among women into account.

From a public health perspective, these findings may also have implications for dietary accessibility. In many settings, protein sources other than red meat, such as legumes, dairy products, eggs, and plant-based foods, may be more accessible and economically feasible^54^. Global analyses have suggested that dietary patterns with reduced red meat consumption and a greater proportion of plant-based foods may not only improve health outcomes but also be more affordable and environmentally sustainable in many regions^55^.

This study has some limitations. First, iron supplementation provided an additional source of iron in both groups, so evaluating the single effect of the dietary intervention was not feasible; therefore, the presented results should be considered in this context. The sample size may be insufficient to identify genuine differences associated with the intervention, as suggested by a post-hoc power analysis; therefore, the absence of a physiological or clinical effect related to the intervention cannot be conclusively ruled out. Future studies may require a large sample size to detect the smallest changes. Since hepcidin or IL-6 levels were not elevated at baseline, as initially hypothesized, the primary outcome was reconsidered and modified before statistical analysis to change in serum iron rather than in hepcidin levels, as originally proposed. Although this may raise a methodological concern, we propose that serum iron provides a more direct and relevant measure in our study population, so results should be considered in this context. More specific details about menstrual history or reproductive status, such as the last menstrual period, number of menstrual pads used, or presence of spotting in underwear, were not collected, which could provide more information about how period-related bleeding could affect current iron status. Although different strategies for monitoring treatment adherence were implemented, as in any dietary intervention study, there is a chance of error in dietary assessment and follow-up. Finally, the intervention length may not have been long enough to demonstrate actual differences. Although previous studies have shown that measurable improvements in iron biomarkers, particularly serum ferritin, can occur after approximately 6–8 weeks of oral iron supplementation or dietary intervention^22,56^, a recent meta-analysis of 10 studies concluded that interventions that included red meat consumption did not affect serum ferritin concentrations, unless they lasted longer than 8 weeks^57^, while complete restoration of iron stores often requires longer treatment periods. Therefore, certain perspectives deserve attention for future research. Studies that focus on postmenopausal women may clarify how reproductive status influences ID. Longer, objectively measured dietary interventions are recommended to explore physiological changes related to iron metabolism and diet, and to evaluate circulating vitamin B12 and folate concentrations to identify any interactions with these micronutrients regarding IDNA and obesity.

## Conclusion

In women with obesity and IDNA, a high-protein diet including red meat did not confer additional benefits for iron status replenishment compared to other dietary iron sources when combined with oral iron supplementation. Our findings suggest that oral iron supplementation has a predominant effect on iron status normalization, regardless of the dietary sources of iron consumed; this provides practical insights suggesting that iron repletion can be achieved solely by supplementation, even in women with obesity who restrict red meat from their diets. While body weight loss and fat mass reduction may play a supportive role in regulating iron metabolism, further research is necessary to confirm their long-term impact.

## Electronic Supplementary Material

Below is the link to the electronic supplementary material.

Supplementary Material 1

## Data Availability

The data that support the findings of this study are available upon request to the corresponding author. The request will be made via email, where the corresponding author will provide a link granting access to the de-identified database of the study, which is deposited in an institutional repository.
