# Supplementary material for: Energy restriction and iron supplementation improve iron status in women with obesity regardless of red meat consumption: a randomized controlled trial
Source: Sci Rep. 2026 May 21;16:23258. doi: 10.1038/s41598-026-53056-8 (PMC13402706; doi:10.1038/s41598-026-53056-8)
Supplement: Supplementary file 1 — Supplementary Material 1 [file 41598_2026_53056_MOESM1_ESM.pdf]

ENERGY RESTRICTION AND IRON SUPPLEMENTATION IMPROVE IRON STATUS IN WOMEN WITH OBESITY REGARDLESS OF RED MEAT CONSUMPTION: A RANDOMIZED CONTROLLED TRIAL.

Supplementary Table S1. Composition of the intervention diets.

	RM			WR		
Energy (kcal)	1600	1800	2000	1600	1800	2000
Protein (%)	20	20	22	20	20	22
CHO (%)	50	50	50	50	50	50
Fiber (g)	25	25	25	25	25	25
Fat (%)	30	30	28	30	30	28
Saturated fat (%)	<7	<7	<7	<7	<7	<7
Trans fat (%)	<1	<1	<1	<1	<1	<1
Cholesterol (mg)	<250	<250	<250	<200	<200	<200
Iron (mg)	18	18	18	18	18	18
Vitamin C (mg)	75	75	75	75	75	75

Abbreviations: CHO, carbohydrates; RM, low-calorie, high-protein diet with red meat; WR, low-calorie, high-protein diet without red meat.

Supplementary Table S2. Iron status indicators and biochemical markers by Intention-to-Treat analysis.

Variables	RM			WR			p[b]	p[c]	p[d]	η^2
	Baseline n = 26	Final n = 26	p[a]	Baseline n = 26	Final n = 26	p[a]				
Serum iron (µg/dL)	59.9 ± 31.5	72.1 ± 32.5	0.13	69.9 ± 30.1	80.1 ± 44.0	0.31	0.22	0.08	0.87	0.001
Ferritin (ng/mL)	26.1 ± 21.2	36.6 ± 27.6	0.004	22.9 ± 11.3	31.8 ± 19.0	0.004	0.45	< 0.001	0.70	0.003
UIBC (µg/dL)	276.4 ± 66.3	254.0 ± 61.1	0.04	264.2 ± 55.5	238.9 ± 50.7	0.006	0.35	< 0.001	0.81	0.001
Transferrin (mg/dL)	314.5 ± 48.6	301.2 ± 39.0	0.05	312.8 ± 44.0	290.7 ± 33.6	< 0.001	0.57	< 0.001	0.28	0.02
Hepcidin (ng/mL)	2.2 (0.9-6.7)	5.1 (2.4-10.4)	0.11	4.6 (2.1-8.3)	6.2 (3.9-9.5)	0.02	0.30	0.003	0.97	0.001
TIBC (µg/dL)	335.2 ± 47.6	326.4 ± 39.5	0.09	334.1 ± 49.0	319.0 ± 38.2	0.01	0.67	0.004	0.53	0.008
Transferrin Sat. (%)	18.6 ± 10.7	22.6 ± 11.0	0.08	21.2 ± 9.5	25.0 ± 12.7	0.16	0.32	0.02	0.95	0.001
Hemoglobin (g/dL)	13.5 ± 1.8	13.6 ± 1.3	0.52	14.4 ± 0.9	14.1 ± 0.7	0.18	0.06	0.79	0.19	0.03
MCV (fL)	86.1 ± 9.8	88.0 ± 6.4	0.81	88.1 ± 6.5	88.0 ± 4.7	0.84	0.60	0.14	0.09	0.05
RDW (%)	15.0 ± 1.6	15.2 ± 2.5	0.42	14.3 ± 1.4	14.2 ± 1.3	0.38	0.06	0.68	0.27	0.02
Glucose (mg/dL)	99.5 ± 9.9	94.7 ± 10.0	0.005	96.9 ± 9.6	95.8 ± 7.7	0.35	0.76	0.004	0.07	0.06
Triglycerides (mg/dL)	122.7 (99.5-164.9)	118.6 (106.4-156.3)	0.89	142.6 (106.5-175.8)	129.2 (101.1-168.2)	0.08	0.34	0.45	0.35	0.01
TC (mg/dL)	166.4 ± 31.9	167.8 ± 27.3	0.74	168.6 ± 35.3	167.9 ± 30.1	0.86	0.88	0.90	0.72	0.003
LDL-C (mg/dL)	110.5 ± 27.2	114.5 ± 22.4	0.28	111.0 ± 30.7	116.4 ± 28.1	0.23	0.03	0.10	0.82	0.001
HDL-C (mg/dL)	42.2 ± 10.5	43.8 ± 9.5	0.33	41.2 ± 7.6	44.1 ± 10.2	0.001	0.42	0.01	0.36	0.01
Insulin (µU/mL)	10.2 (7.5-22.1)	12.5 (4.0-21.2)	0.58	12.5 (8.8-15.9)	15.4 (8.2-24.2)	0.27	0.45	0.95	0.47	0.01

Leptin (ng/mL)	53.4 (34.7-71.8)	50.4 (27.2-71.6)	0.24	60.1 (46.7-92.4)	42.0 (35.9-66.3)	< 0.001	0.20	0.005	0.06	0.08
CRP (mg/L)	5.6 (3.1-28.6)	5.4 (2.0-13.0)	0.54	6.5 (3.8-10.3)	7.2 (2.7-9.9)	0.74	0.41	0.17	0.58	0.006
ALT (UI/L)	21.4 (17.5-27.8)	22.8 (16.8-31.2)	0.68	21.1 (16.3-30.0)	20.6 (16.2-26.1)	0.38	0.26	0.80	0.41	0.01
AST (UI/L)	19.7 (17.1-25.3)	18.8 (17.5-23.8)	0.78	19.1 (17.3-25.3)	18.0 (15.3-22.6)	0.37	0.36	0.46	0.72	0.003
Albumin (g/L)	39.0 ± 2.6	39.0 ± 3.3	0.95	39.8 ± 1.8	38.9 ± 3.3	0.20	0.59	0.39	0.73	0.01
Creatinine (mg/dL)	0.6 ± 0.1	0.7 ± 0.1	0.40	0.7 ± 0.1	0.7 ± 0.1	0.77	0.95	0.39	0.60	0.005
Urea (mmol/L)	4.1 ± 0.8	4.1 ± 1.0	0.63	4.2 ± 0.8	4.5 ± 0.9	0.12	0.50	0.14	0.39	0.01
Uric acid (μmol/L)	351.8 ± 75.2	290.8 ± 78.9	0.002	341.1 ± 79.0	297.3 ± 80.1	0.006	0.90	< 0.001	0.44	0.01
MDA (nmol/mL)	1.0 (0.0-1.6)	0.01 (0.0-0.001)	0.005	0.01 (0-1.2)	0.01 (0.0-0.4)	0.36	0.10	0.98	0.88	0.003

The intention-to-treat analysis included all randomized participants; missing data were handled by multiple imputation. Data are presented as the mean ± standard deviation or median (interquartile range). Statistical differences (baseline vs final) within groups were examined using paired-samples t-tests or Wilcoxon signed-rank tests. Differences between groups, over time, and the interaction between group and time were evaluated by repeated-measures ANOVA. Variables with nonparametric distributions were normalized with log transformation before repeated-measures ANOVA. The effect size was estimated using partial eta squared (η^2). A p-value < 0.05 was considered statistically significant.

Abbreviations: ALT, alanine transaminase; AST, aspartate transaminase; CRP, C-reactive protein; HDL-C, high-density lipoprotein; LDL-C, low-density lipoprotein; MCV, mean corpuscular volume; MDA, malondialdehyde; RDW, red cell distribution width; RM, low-calorie, high-protein diet with red meat; TC, total cholesterol; TIBC, total iron-binding capacity; Transferrin Sat., transferrin saturation; UIBC, unsaturated iron binding capacity; WR, low-calorie, high-protein diet without red meat.

[a]paired-samples t-test or Wilcoxon signed-rank test.

[b]Repeated-measures ANOVA (group).

[c]Repeated-measures ANOVA (time).

[d]Repeated-measures ANOVA (group-time interaction; primary hypothesis).

Supplementary Table S3. Dietary assessment of participants.

	RM			WR				
	Baseline n = 22	Final n = 22	p†	Baseline n = 23	Final n = 23	p†	p*	η^2
Energy (kcal)	1824 ± 763	1425 ± 475	0.05	2079 ± 744	1498 ± 392	0.003	0.48	0.01
CHO (%)	49 ± 8	48 ± 14	0.73	48 ± 10	49 ± 8	0.79	0.66	0.005
Fiber (g)	0.9 ± 1.5	3.2 ± 3.5	0.009	1.7 ± 2.4	3.1 ± 3.2	0.04	0.44	0.01
Fat (%)	34 ± 6	32 ± 9	0.44	35 ± 8	32 ± 10	0.21	0.75	0.002
Saturated fat (%)	11 ± 4	10 ± 5	0.29	11 ± 4	10 ± 5	0.13	0.66	0.004
Trans fat (%)	0.16 ± 0.15	0.13 ± 0.17	0.59	0.15 ± 0.17	0.09 ± 0.13	0.25	0.65	0.005
Cholesterol (mg)	251 ± 140	172 ± 82	0.02	260 ± 126	199 ± 74	0.04	0.67	0.004
Protein (%)	18 ± 8	22 ± 8	0.04	19 ± 5	22 ± 5	0.04	0.72	0.003
Protein (g/kg_{ibw})	1.5 ± 0.6	1.8 ± 0.6	0.76	1.4 ± 0.6	1.5 ± 0.4	0.07	0.31	0.024
Iron (mg)	8.2 ± 6.0	9.6 ± 4.5	0.59	9.8 ± 5.1	9.9 ± 3.3	0.07	0.41	0.015
Vitamin B_{12} (µg)	4.0 ± 6.8	3.0 ± 1.5	0.50	2.9 ± 1.3	3.1 ± 2.1	0.55	0.40	0.016
Folate (µg)	145 (99-227)	241 (184-384)	0.13	199 (112-374)	237 (203-324)	0.07	0.69	0.004

Vitamin C (mg)	36.3 (11.2-134.4)	93.3 (48-251)	0.02	65.1 (24.1-119.3)	122.7 (35-193)	0.07	0.28	0.027

Data are shown as mean ± standard deviation; statistical differences after follow-up within groups were tested by t-test for related samples (†), while the effect of interventions on changes observed was explored with repeated measures ANOVA (*), and the effect size was estimated by partial eta squared (η^2). A p-value <0.05 was considered significant.

Abbreviations: CHO, carbohydrates; kg_{ibw}, kilograms of ideal body weight; RM, low-calorie, high-protein diet with red meat; WR, low-calorie, high-protein diet without red meat.

Notes:
